# Lymph Node Metastases from Non-Melanoma Skin Cancer of the Head and Neck †

**DOI:** 10.3390/cancers15174201

**Published:** 2023-08-22

**Authors:** Francisco Civantos, Zachary M. Helmen, Patrick J. Bradley, Andrés Coca-Pelaz, Remco De Bree, Orlando Guntinas-Lichius, Luiz P. Kowalski, Fernando López, Antti A. Mäkitie, Alessandra Rinaldo, K. Thomas Robbins, Juan P. Rodrigo, Robert P. Takes, Alfio Ferlito

**Affiliations:** 1 Department of Otolaryngology-Head and Neck Surgery, University of Miami Miller School of Medicine, Miami, FL 33136, USA; fcivanto@med.miami.edu (F.C.); zachary.helmen@jhsmiami.org (Z.M.H.); 2 Department of Otorhinolaryngology-Head and Neck Surgery, Nottingham University Hospitals, Queens Medical Centre Campus, Nottingham NG7 2UH, UK; 3 Department of Otolaryngology, Hospital Universitario Central de Asturias, University of Oviedo, ISPA, IUOPA, CIBERONC, 33011 Oviedo, Spain; acocapelaz@yahoo.es (A.C.-P.); fernandolopezphd@gmail.com (F.L.);; 4 Department of Head and Neck Surgical Oncology, University Medical Center Utrecht, 3508 GA Utrecht, The Netherlands; 5 Department of Otorhinolaryngology, Institute of Phoniatry/Pedaudiology, Jena University Hospital, 07747 Jena, Germany; 6 Department of Head and Neck Surgery and Otorhinolaryngology, A.C. Camargo Cancer Center, Sao Paolo 01509-900, Brazil; 7 Head and Neck Surgery Department, University of São Paulo Medical School, Sao Paulo 05403-000, Brazil; 8 Department of Otorhinolaryngology-Head and Neck Surgery, Research Program in Systems Oncology, Faculty of Medicine, University of Helsinki and Helsinki University Hospital, FI-00029 HUS Helsinki, Finland; antti.makitie@helsinki.fi; 9 ENT Unit, Policlinico Città di Udine, 33100 Udine, Italy; 10 Department of Otolaryngology Head and Neck Surgery, School of Medicine, Southern Illinois University Carbondale, Carbondale, IL 62901, USA; 11 Department of Otorhinolaryngology-Head and Neck Surgery, Radboud University Medical Centre, 6525 GA Nijmegen, The Netherlands; 12 Coordinator of the International Head and Neck Scientific Group, 35100 Padua, Italy

**Keywords:** non-melanoma skin cancer, squamous cell carcinoma, Merkel cell carcinoma, eccrine cell carcinoma, neck metastases, neck dissection, sentinel lymph node biopsy

## Abstract

**Simple Summary:**

Skin cancer, particularly non-melanoma skin cancer, is the most common malignancy in the world. There are both common and uncommon types that receive treatment every day. Despite their commonality, the management of each is not perfectly defined in the scientific literature. Many require surgical removal, but the management of regional metastasis (such as lymph nodes in the neck) may or may not require surgical removal, or even anything beyond observation. Further complicating matters, some may have microscopic regional metastases that cannot be detected with a physical exam or imaging. This article seeks to summarize the current literature on this topic and to offer specific insight on how to manage non-melanoma skin cancer that has migrated away from the primary site to the regional lymph nodes.

**Abstract:**

Non-melanoma skin cancer (NMSC) represents the most common malignancy in the world, comprising exceedingly common lesions such as basal cell carcinoma (BCC) and cutaneous squamous cell carcinoma (cSCC) and rare lesions such as Merkel cell carcinoma. Risk factors are widely recognized and include ultraviolet (UV) light exposure, radiation exposure, immunosuppression, and many others. As a whole, survival and functional outcomes are favorable, but each histopathological subtype of NMSC behaves differently. Treatment regimens for the primary site usually include wide surgical excision and neck dissection in cases of clinically involved metastatic lymph nodes. The elective management of draining nodal basins, however, is a contested topic. Nearly all subtypes, excluding BCC, have a significant risk of lymphatic metastases, and have been studied with regard to sentinel lymph node biopsy (SLNB) and elective neck dissection. To date, no studies have definitively established a true single standard of care, as exists for melanoma, for any of the NMSCs. As a result, the authors have sought to summarize the current literature and identify indications and management options for the management of the cervical lymphatics for each major subtype of NMSC. Further research remains critically necessary in order to develop complete treatment algorithms.

## 1. Introduction

Non-melanoma skin cancer (NMSC) represents the most common category of malignancy in the world [1]. Major predisposing factors for NMSC include chronic UV light exposure, childhood sunshine exposure, fair skin, ionizing radiation, immunosuppression, and genetic mutations [2]. While common subtypes such as basal cell carcinoma (BCC) and cutaneous squamous cell carcinoma (cSCC) of the head and neck are treated by a variety of specialties, other NMSCs such as Merkel cell carcinoma (MCC), eccrine cell carcinoma, and invasive squamous cell carcinomas with high-risk features or advanced stage are either exceedingly rare or difficult to treat, and will frequently require multidisciplinary treatment including advanced head and neck surgical expertise. Management of the primary tumor is certainly important, and represents the first step, but neglecting to manage lymph node metastasis can have detrimental side effects. While some tumor types almost never require addressing nodal basins, sentinel lymph node biopsy (SLNB), neck irradiation, or neck dissection may be recommended in other NMSC [3]. This review intends to focus on the management of lymph node metastasis of head and neck NMSCs.

## 2. Management of Lymph Node Metastases of Non-Melanoma Skin Cancer (NMSC) of the Head and Neck

### 2.1. Cutaneous Squamous Cell Carcinoma—Epidemiology and Risk Factors

Cutaneous squamous cell carcinoma (cSCC) is a malignant tumor arising from keratinocytes. It is the second most common cutaneous malignancy, comprising 10–20% of all cutaneous malignancies [4,5]. The five-year overall survival is extremely high, but it is not without risk of local tissue destruction, metastases, morbidity, and mortality [6]. Survival drops dramatically when metastases occur [7]. Risk factors for the development of cSCC include sunlight, indoor tanning, fair skin pigmentation, scars or chronic wounds, immunosuppression, genetic defects, and a previous history of actinic keratosis [8,9,10,11,12,13,14,15]. Approximately 55% of all cutaneous SCC occurs on the head and neck and frequently involves the extensor surfaces of the hands and forearms (18%) [16]. Recent studies have suggested that high-risk subtypes are more common on the face than low-risk subtypes [17]. The incidence of cSCCs has increased progressively from 50% to 300% in the last thirty years, and this is expected to double by 2030 [18,19]. The estimated risk of developing a cSCC is 7% to 11% in the Caucasian population (9–14% in men and 4–9% in women) [20,21].

Though still not the most common NMSC, cSCC represents the majority of NMSC mortality, partially secondary to its proclivity for regional lymph node metastasis [22]. Cutaneous SCC represents a very heterogeneous group of tumors, with very different behaviors. In this article, we are less interested in superficial disease, in situ (Bowen’s disease) or minimally invasive lesions, for which there is little concern regarding lymphatic metastases. There are specific potential harbingers of aggressive behavior leading to regional nodal metastasis, distant metastasis, and recurrence, including tumors more than 2 cm in diameter [23,24,25], poor histologic differentiation [26], depth of invasion more than 4 mm [27], Clark level IV–V [26], perineural involvement [28], recurrence [26], peri-parotid or intra-parotid and peri-auricular location [29], tumor budding [30], immunosuppression, extracapsular spread, and lymph node ratio [31]. Perineural invasion is the worst overall pathological indicator, carrying approximately a 50% risk of recurrence. Within this category, gross perineural invasion of large nerves is the worst indicator of locoregional recurrence [4]. Based on the study by Brantsch et al., tumor thickness may be the strongest predictor of regional lymph node metastasis [29]. This was confirmed in a large meta-analysis [27]. What is important to note, however, is that most of these studies are related to cSCC of all subsites in the body. Those specifically focused on the head and neck have made varying conclusions regarding the relative importance of each risk factor, some even contradictory [22]. The diametrically opposed conclusions in studies specifically for head and neck cSCC have been attributed to the fact that these are small studies with a high risk for type 1 and type 2 error (inappropriate interpretation of random variation in small studies leading to false positive or false negative assumptions).

There is compelling evidence for the importance of the immune system in the pathogenesis of skin cancer, but the underlying mechanisms and their role in patient prognosis have been poorly analyzed [32,33]. Santos-Juanes et al. studied the interaction of lectin-like transcript 1 (LLT1) with CD161, which inhibits the activation of natural killer cells, favoring tumor progression. LLT1 overexpression is a significant predictor of risk for nodal metastasis and disease-specific survival in cSCC patients [34]. Other biomarkers that appear to correlate with the risk of developing lymphatic metastases in sSCCs include focal adhesion kinase (FAK) and cortactin overexpression and high PD-L1 expression [35,36].

High-risk cSCC with respect to nodal metastases was defined by Burton et al. as a cSCC that is staged as N0, extends beyond the basement membrane, and has high-risk features associated with sub-clinical metastasis. These features include depth of invasion (>2 mm), high grade histological differentiation, high-risk anatomic location (face, ear, pre/post auricular, genitalia, hands, and feet), perineural invasion, recurrence, multiple cSCC tumors, and immunosuppression [37]. The largest prospective studies involving SLNB in high-risk cSCC of the head and neck only found the number of high-risk features (as compared to specific individual features themselves) to be significant in predicting lymph node metastasis [38]. Studies do reveal metastatic rates of 3–9%, evolving an average of one to two years after initial diagnosis [39]. The anatomic primary site with the highest incidence of lymphatic metastasis is the auricle [40]. Clinical detection is difficult as it is infrequent for cSCC to have clinically palpable lymphatic metastases at the time of diagnosis of the skin lesion, occurring less than 2% of the time in a series of 7000 patients [41]. This rate of metastasis is lower than that generally accepted as indicating a need for prophylactic neck treatment, and, for this reason, there has been a need to identify a higher risk group for the surgical staging of the neck. In the absence of randomized trials, studies have attempted to evaluate and use risk stratification tools, based on the limited available data, using the most important risk factors associated with lymphatic metastases [42,43].

### 2.2. Cutaneous Squamous Cell Carcinoma—Diagnosis, Evaluation, and Sentinel Lymph Node Biopsy

**Figure 1:**
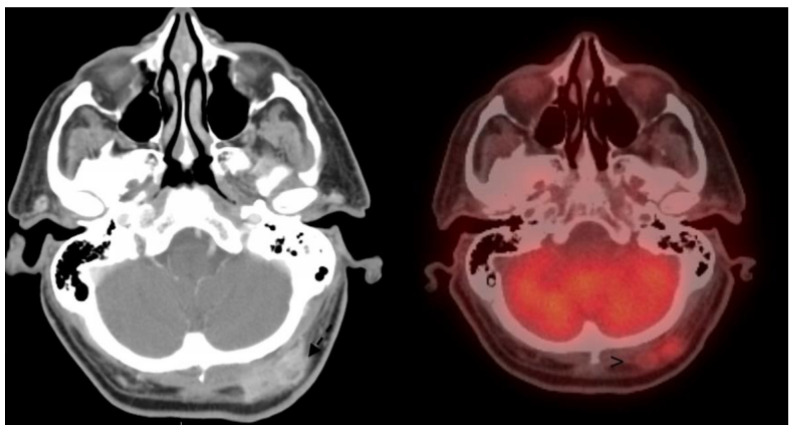
Axial contrasted computed tomography (CT) of the head on (**left**) with involved occipital node from a primary scalp squamous cell carcinoma, with positive fluorine-18-fluorodeoxyglucose positron emission tomography/computed tomography (FDG-PET/CT) as seen on (**right**).

In order to develop risk stratification, evaluation begins with patient history and a physical exam assessing the size and estimated depth and thickness of the lesion by palpation. At the time of evaluation of the concerning primary lesion, imaging can be considered. For more extensive lesions, imaging is effective in evaluating the primary lesion, and can reveal regional and distant disease as well. Similar to the imaging principles used for other malignancies, contrast-enhanced computed tomography (CT) is preferred for the evaluation of bony involvement while magnetic resonance imaging (MRI) is preferred for soft tissue evaluation and perineural involvement [44]. Positron emission tomography/computed tomography (PET/CT) can be useful in the evaluation of nodal or distant disease as clinically indicated, particularly as an adjunct to contrasted CT or MRI (see Figure 1) [25].

At the time of the initial workup of cSCC, the consideration of the nodal drainage is necessary. Imaging followed by ultrasound-guided fine needle aspiration (FNA) or core biopsy should be pursued due to the high risk of false positive clinical examination alone [45]. In those with negative biopsy but persistent suspicious nodal disease, repeat evaluation, imaging, core biopsy, or surgical excisional biopsy should be considered [25]. In those without obvious adenopathy on physical examination or imaging, i.e., the N0 patient, occult metastasis should be considered, and the minimum is close observation—the watchful waiting approach—with patient education and serial physical examination with or without ultrasound-guided FNA. The primary way this can be evaluated more definitively in the high-risk patient, however not clearly defined, is with SLNB [46]. Identifying those with positive metastatic nodal disease allows the surgeon to guide early treatment accordingly to either observation or neck dissection and/or radiation therapy (RT). That said, it is not a perfect intervention and false negatives have been reported between zero and twelve percent of the time [47,48]. False negatives can be a result of sampling error, pathological analysis error, or unexpected patterns of lymph node drainage in patients with prior surgery [47,48]. Despite its obvious therapeutic benefit, data for all subsites of cSCC have shown that those with positive SLNB biopsy are more likely to have postoperative recurrence, metastases, and decreased disease-specific survival [47,48,49,50,51]. It is unclear if this represents identifying more aggressive disease that would have behaved more aggressively with or without positive SLNB, and if there are improved patient outcomes with this intervention.

Currently, there is no set criterion for identifying those patients with cSCC who would benefit from SLNB. A recent large systematic review by Lubov et al., including over 8000 patients, made recommendations—deemed reasonable in the absence of high-level data—regarding selection criteria [52]. They outlined the state of immunosuppression and depth of a tumor ≥6 mm or beyond subcutaneous fat as major criteria. Minor criteria included perineural invasion, lymphovascular invasion, or poor histologic differentiation. Their recommendation was to offer SLNB to those with either two major criteria or those with one major and two minor criteria [52]. It is important to note that these conclusions are based on studies performed on cSCC both inside and outside the head and neck region, and that there are no current prospective trials that have studied this specifically for the head and neck. Furthermore, the recommendations are based on observational data, and reasonable assumptions, not a demonstration of improved survival in randomized trials. It should also be noted that excisional or large biopsies would be needed, including the sampling of the interface of the tumor with surrounding tissue, before definitive resection with wide margins. This would provide as much pathological information as possible to achieve stratification based on the high-risk pathological features mentioned above as small biopsies may not provide pathological data regarding the depth of invasion, perineural invasion, and lymphovascular invasion, or other high-risk features such as tumor budding, usually defined as isolated single cancer cells or clusters of up to four cancer cells located at the invasive tumor front.

Critics of adoption of the SLNB approach in cSCC argue that many patients do not require it; thus, it should not be standard for all patients. Other studies have attempted to categorize these patients and identify risk factors for occult metastases. In designing a study for SLNB for cSCC, the downside is that if the definition of patients “at risk” is defined too broadly, many patients would undergo neck surgery and very few positive nodes would be identified. Additionally, SLNB is not always practical in patients deemed at risk because of the extremely advanced stage at the primary site (i.e., T4) without clinic nodal disease. Such tumors may be invading deeply into the parotid or neck, altering lymphatic drainage, and requiring a nodal dissection to resect the primary itself [47]. Moreover, many cSCCs can be excised under local anesthesia, while SLNB is generally preformed under general anesthesia.

To help identify those who would need SLNB, Durham et al. [47] identified 53 patients with cSCC at high risk for nodal metastasis using risk factors from the National Comprehensive Cancer Network (NCCN): Breslow depth of 2 mm of more or Clark level of IV or V; rapid growth; locally recurrent; occurrence in a prior radiation or chronic inflammation and/or ulcer site; perineural invasion; angiolymphatic invasion; immunosuppression; size of 1 cm or more on the cheek, forehead, scalp, neck, or 0.6 cm or more on the face mask area; and poorly differentiated histologic pattern [25]. As a result, they identified SLNB positivity in 11.3% of cases. Furthermore, with tissue processing and immunohistochemistry, they revealed an adjusted SLNB rate of 15.1%. Risk factors for the presence of nodal disease included angiolymphatic invasion, perineural invasion, and clinical size [47]. They concluded that full sectioning and immunohistochemistry are important for the accurate diagnosis and guidance of treatment. This would appear to represent a very useful risk stratification given the 15.1% rate of SLN positivity.

A prospective study by Gore et al. began with offering wide excision of the tumor and concurrent SNLB for clinically high-risk primary or recurrent cSCC [48]. Those who were identified as high-risk after excision based on pathology were offered secondary SNLB and possible wider excision if appropriate. Inclusion criteria were (1) tumor size >2 cm; (2) invasion into subcutaneous fat or tumor thickness >5 mm; (3) poorly differentiated tumor; (4) perineural invasion; (5) lymphovascular invasion; (6) local recurrence in the setting of adequate prior resection margins; (7) ear or lip location; (8) immunocompromise; and (9) carcinoma in a preexisting scar. They found that 14% of patients had subclinical nodal metastasis. Strong predictors of nodal metastasis included the number of high-risk tumor factors present and the pathologic diagnosis of perineural invasion, and lymphovascular invasion [48]. This evidence suggests that patients presenting with numerous high-risk tumor factors should be considered for SLNB. Several additional studies with smaller numbers of patients have been reported for SLNB in cSCC, but Gore’s analysis of 57 patients remains the largest to date.

A different approach by Haisma et al. sought to identify risk factors for a much larger cohort by retrospectively evaluating primary tumors for histopathological risk factors that predisposed patients to the development of lymph node metastasis [53]. The study analyzed over 300 patients with cSCC of the head and neck, identifying lymph node metastasis in 55 patients (16.4%). Multivariate analysis identified the following risk factors for the development of nodal disease: location on ear, tumor diameter >50 mm, moderate or poor differentiation, and tumor thickness >2 mm [53]. There was, however, a correlation between tumor thickness and tumor diameter in the study. In contrast to the above study by Durham et al. [47], immunosuppression had no significant influence on the development of metastasis. This is also in contrast to other studies [26,27,29]. This difference in result for immunosuppressed patients was attributed by Haisma et al. to possible earlier detection in these specific patients given their frequent follow up and exposure to the medical system, and it was proposed that the lesions in their study were high-risk but earlier in stage. Regardless of the differences between studies, it can be inferred that SLNB should be considered in patients presenting with the risk factors identified via multivariate analysis.

### 2.3. Cutaneous Squamous Cell Carcinoma—Treatment and Staging

The type of treatment of the primary tumor also appears to have an impact on the development of metastases. Thus, improving local tumor control, Mohs surgery appears to reduce the frequency of regional metastatic disease and may confer a survival advantage even for those patients who develop nodal spread, as evidenced by postsalvage disease-specific survival outcomes [54].

There are two main staging systems for cSCC in widespread clinical use: the American Joint Committee on Cancer (AJCC) eighth edition staging system and the Brigham and Woman’s Hospital (BWH) staging system (Table 1) [55,56,57]. There are many similarities, but the systems have been compared in terms of staging accuracy and other metrics. BWH was found to be superior in predicting nodal metastasis and disease-specific death, but there was no difference in predicting local recurrence or overall survival [57]. BWH was also found to identify those with high-risk malignancies in a smaller subset of all patients, limiting inappropriate upstaging. Most of the important risk factors mentioned in the paragraph above are incorporated into these staging systems, but, as of yet, we do not have data, retrospective or randomized, to firmly recommend SLNB or elective neck dissection for particular stages of disease. Additionally, the standard for SLNB in N0 cSCC of the head and neck remains vague and dependent on the surgeon and institutional preference. We also cannot comment which of these staging systems functions better for patient selection for SLNB. Currently, the best method for addressing cervical lymphatics is combining the approaches in the papers by Durham et al. [47], Gore et al. [48], and Haisma et al. [53]. A table has been created summarizing these studies and their associated risk factors (Table 2). This must also take into account the patient’s overall condition and co-morbidities, particularly if a positive SLNB results in converting the surgery from a procedure under local with sedation to a procedure under general anesthesia. Together with the patient, the surgeon must try to arrive at a reasonable decision. It is important to note that this summary (Table 2) and these three studies are not meant to represent an all-inclusive recommendation or meta-analysis. Instead, it is intended to serve as an example of how the analysis of larger studies on the topic may guide the thought process required in surgical decision-making. It is important in future to acquire prospective data to better guide therapy in this area.

Finally, the question remains as to whether SLNB confers only staging accuracy versus actual benefit to the N0 patient. To date, there are only a few studies that comment on this [58,59,60]. Xiao et al. found occult metastasis in the parotid and neck in selected high-risk cSCC to be 20% (six patients) and 16% (five patients), respectively [58]. Those who underwent elective neck dissection and parotidectomy had superior disease-free and disease-specific survival as compared to those who underwent parotidectomy alone or observation [58]. Cannon’s study specifically addressed those with perineural skull base invasion, but without evidence of lymph node metastasis. Those treated with elective neck dissection were found to have improved five-year disease-free survival and overall survival in addition to reduced regional recurrence [59]. An opposing conclusion comes from Amit et al. [60]. This study included nearly 1000 patients without evidence of nodal disease, some undergoing elective neck dissection, others undergoing observation. It should be noted that this study is limited in its retrospective nature. Contrasting to Cannon et al. and Xiao et al., they showed noninferior survival rates in the observation group. The first two studies present evidence to consider the staging and treatment of the cervical lymphatics for high-risk cSCC; however, Amit posits a role for observation. The latter was an extensive study, though not restricted to the head and neck region. Nonetheless, it is clear that the literature is not yet complete on this topic for all patients. SLNB would allow the application of therapeutic neck treatment to lower-stage patients with occult neck disease, while sparing the morbidity of neck dissection for those who have negative sentinel nodes.

It should be remembered that SLNB Is an evaluation and staging technique, but not a treatment. In terms of treatment, a discussion of the treatment options for the primary tumor site is beyond the scope of this review which is focused on the management of lymph node metastases. As a result, the remainder of this section will focus on treating lymph node metastases in cSCC. Nodal involvement significantly increases the risk of recurrence and mortality [43,53,61]. The treatment modality of choice is surgery, as multiple studies have established worse outcomes in those receiving RT alone [62,63]. The extent of neck dissection is debated at this time, but recommendations from one study will be outlined below [64].

Dissimilar from some other subtypes of NMSC, for SCC, the parotid is an important structure to consider in evaluating lymph node metastasis as it is the most common site of metastasis [64]. Those with parotid nodes have been found to have a high risk for both clinical and occult neck involvement [65]. One study identified parotid involvement in 60–82% of those with nodal disease [66]. An additional complicating factor is atypical lymphatic drainage, which eventually can be identified by lymphoscintigraphy. Data extrapolated from melanoma research have quoted drainage outside of the expected anatomical pattern up to 30% of the time [67]. In the setting of established nodal or parotid disease, superficial or total parotidectomy with facial nerve preservation and modified radical neck dissection is suggested by some authors [25,64]. However, for cases where there is parotid disease but no overt neck disease, guidelines are still being developed. Rotman et al. recently performed a systematic review and meta-analysis, finding the presence of occult cervical nodal disease to be 22.5% in patients with cutaneous SCC metastatic to the parotid, thus necessitating elective neck dissection [68]. For a similar study of patients in this subcategory, Vauterin et al. reported that the incidence of occult metastasis is quoted at 36% [69]. A large study out of Sydney, Australia, associated neck levels of occult metastasis with areas of the primary lesion. Their recommendations, in addition to parotidectomy, included selective neck dissection of levels I–III for facial primaries, levels II–III for anterior scalp and external ear, and levels II–V for posterior scalp and neck primaries [70]. In those with established neck metastasis and risk for occult parotid metastasis but no clinical parotid disease, the parotid can be either surgically excised prophylactically, or radiated at the time of adjuvant RT (in order to prevent unnecessary risk to the facial nerve) [64]. Traditionally, patients without parotid disease but with neck disease were managed by excising levels I–V. With a better understanding of drainage pathways, however, selective neck dissection (particularly for those with planned adjuvant RT) can be considered [71]. For those with both parotid and neck disease, parotidectomy and comprehensive neck dissection (level I–V) have been suggested, as those in the clinical category have been found to have a 35% rate of positive nodes in level V [69]. Finally, atypical nodal basins must be carefully examined and considered for dissection including retroauricular, suboccipital, and external jugular nodes (see Figure 1). Indications for adjuvant RT to the primary site include a positive margin that cannot be re-resected, perineural invasion, muscle/cartilage or bony involvement, and nodal involvement [25]. RT indications for the elective postoperative treatment of undissected lymphatics include recurrences, poorly differentiated tumors, tumors over 3 cm, and large infiltrative, ulcerative SCC [72]. A brief comparative summary is shown in Table 3.

In considering the above-mentioned indications, adjuvant RT has been associated with better locoregional control and disease-free survival in some studies with appropriately selected patients [73,74,75,76]. A separate study, however, concluded no difference between these two metrics, possibly suggesting no need for adjuvant RT [77]. There are also mixed results for overall survival [77,78]. It is highly likely that these differences are explained by differences in the stage and extent of disease, and the pathological risk factors between the studies. Nevertheless, NCCN guidelines currently recommend adjuvant RT in specific circumstances (multiple nodes, one node greater than 3 cm in size, extracapsular extension, or incomplete excision) following surgical lymphadenectomy [25]. Forgoing adjuvant RT is considered a reasonable alternative in those with a solitary node less than 3 cm without extracapsular extension [25,64]. Systemic therapy has been studied, including cytotoxic methods, EGFR inhibitors, and monoclonal antibodies. However, given a lack of prospective, comparative studies, the NCCN does not have a formal recommendation on routine use [25]. A recent trial showed no oncologic benefit (locoregional control or survival) with the addition of weekly carboplatin in the setting of postoperative RT, even in those with high-risk cSCC [79]. Immunotherapy is also a burgeoning topic within cSCC, but is outside the scope of this focused review. Many immunotherapy studies focus on high-risk or recurrent patients, studying agents such as cemiplimab (PD-1 blockade), an neoadjuvant immunotherapy, amongst others [80,81] This further underscores the need for surgical resection, identification of occult metastases, removal, and adjuvant RT. Future treatment paradigms will certainly involve a combination of these treatment modalities, but with a larger focus on gene expression [82,83]. In fact induction immunotherapy is now an emerging option for patients with advanced disease as an alternative to very morbid resections [80,81]. Regardless of treatment modality, surveillance is essential to ensure the swift recognition of recurrence, locoregional metastasis, or second primary.

### 2.4. Merkel Cell Carcinoma

Previously known as trabecular carcinoma, Merkel cell carcinoma (MCC) is a rare but very aggressive cutaneous high-grade tumor of neuroendocrine origin with poor prognosis, particularly when presenting at advanced stages [84]. Approximately 2500 cases per year are diagnosed in the United States [85]. Pathogenesis is similar to other NMSC with sun exposure being a major risk factor, but a unique pathogenic mechanism involves the presence of Merkel cell polyomavirus in at least 60% of cases. Feng et al. [86]. identified this novel polyomavirus in 80% of their MCC tumor tissue samples in 2008 [87]. When compared with polyomavirus-negative MCC, those with Merkel cell polyomavirus have had a better prognosis [88]. Those that do not have polyomavirus genetic sequences are thought to be related to sun exposure, and would generally have typical genetic signatures related to ultraviolet exposure on next generation sequencing data [89]. There have been multiple studies displaying a component of immunosuppression in the pathogenesis of this malignancy, and others that show worse disease-specific survival in immunosuppressed individuals [90,91]. Micro RNAs are thought to be a pathologic mechanism as well [92]. Additionally, MCC of the lip, as compared to other head and neck subsites, portends worse survival overall [93]. Despite its aggressive nature, there are often no clinical features that consistently distinguish it from other benign or malignant masses, about half being diagnosed as benign cysts/lesions originally [87]. Roughly 25 percent to 33 percent of MCC present with nodal involvement [94,95].

Before beginning further discussion on the management of lymph nodes in MCC, it is important to establish that the relative rarity of MCC has limited rigorous prospective studies to establish guidelines. The following discussion draws on the current data, but conclusions are made in small individual studies and meta-analyses.

A discussion of specific microscopic findings of MCC is beyond the scope of this focused review. Nonetheless, some predictors of SLN positivity can be found in the pathology report. Factors associated with sentinel lymph node positivity in some studies have included tumor diameter, tumor thickness, mitotic rate, location of the primary, lymphovascular invasion, and tumor-infiltrating lymphocytes [96,97,98,99]. Additionally, MCC can be classified in the group of “small round blue cell tumors” [100].

The workup of lymph node metastasis for MCC should begin with imaging prior to surgical intervention. CT with contrast is a common modality for most head and neck malignancies but can only detect gross disease. It has proven less useful in MCC due to a propensity for subclinical metastases. Gupta et al. found the sensitivity of baseline imaging for lymph node metastasis to be only 20% [101]. A similar study found the specificity of CT to be 97%, but the sensitivity to be 47%, failing to detect both micrometastases and macrometastases below the size thresholds of positivity using CT criteria [102]. This same study compared imaging modalities of patients with MCC, concluding that fluorine-18-fluorodeoxyglucose PET/CT is more sensitive and equally specific when compared to CT. MRI showed extremely low sensitivity (0%) and favorable specificity (86%) [102].

Multiple studies have been performed to ascertain the utility of PET/CT in assessing nodal disease burden, with varying results regarding sensitivity. The study by Colgan et al. referenced above found the sensitivity of PET/CT to be 83% [102]. Two other analyses found sensitivity to be as low as 10 to 14% [103,104]. However, when comparing studies with less stringent criteria for verifying imaging, including a variety of disease stages, the sensitivity is reported to be 86–100% and specificity 89–100% [105]. Given the wide variability in imaging efficacy, the current NCCN guidelines do not generally recommend imaging for identifying subclinical or regional disease in asymptomatic patients. The recommendation is to order imaging studies as clinically indicated [106]. Additionally, the NCCN suggests that imaging may be indicated to evaluate the possibility that the presumed MCC could be a skin metastasis of a non-cutaneous neuroendocrine carcinoma (i.e., small cell carcinoma of the lung or neuroendocrine carcinoma of the digestive tract), and to screen for secondary skin malignancies in these anatomic areas if the cutaneous origin of the lesion cannot be absolutely confirmed histologically [106]. Nonetheless, subclinical positive disease in the parotid can be identified in MRI or CT, particularly the former, and these modalities are frequently used to look for suspicious nodes that are clinically evident on imaging but not palpable, and that could serve as targets for fine needle aspiration. As for all head and neck cancers, ultrasound can be useful in the cervical area [107,108].

Given that MCC is an aggressive tumor and imaging is not entirely conclusive, the pathological staging of the lymphatics is useful both for establishing treatment and overall prognosis. This can be done with SLNB and classified according to node-negative or node-positive disease [105]. For patients with clinically node-negative disease, identifying micrometastases is important. One large study looked at 375 patients with MCC from 1988 to 2011 and found SLNB to be positive in 31% [96]. Some studies have associated negative SLNB with improved disease-specific survival and overall survival and reduced recurrence [101,109,110,111], while others have shown no significant improvement in prognosis or survival [97,112,113].

Surgical excision of the primary and concurrent SLNB is currently recommended by the NCCN for patients who are fit for surgery with clinically node-negative disease [106]. This recommendation stands despite its unclear benefit on overall survival as it is important for both staging and guiding treatment with either full lymphadenectomy and/or RT. For advanced primary site stage or positive sentinel nodes, further imaging workup should be obtained both to obtain a baseline, but also to assess for metastatic disease and treat accordingly.

Interestingly, there is possibility that SLNB itself offers therapeutic oncologic protection. This has been shown in two larger studies, one showing improved disease-specific survival for those undergoing SLNB [111] and the other showing improved disease-specific mortality and all-cause mortality as well [114]. While an interesting finding, other studies have had opposing conclusions [91,115]. The exact explanation for these differences is currently unknown. The extremely low incidence of the disease limits large prospective trials to offer better conclusions.

In clinically node-positive disease, FNA has proven itself useful in diagnosing both primary, nodal, and metastatic MCC. A study by Righi et al. showed favorable results when using an ultrasound to perform FNA on nodes larger than 6 mm [116]. If needed, open biopsy can be considered. Similar to SLNB, if positive, further imaging and possible subsequent treatment changes should be considered.

In the setting of positive nodal disease of MCC, there have been a multitude of studies outlined in the NCCN guidelines associating regional disease with poor prognosis [106]. Surgical lymphadenectomy and RT as separate modalities have been shown to be associated with improved disease control, time to recurrence, and 3-year relapse survival [101,117]. As discussed multiple times in this focused review, however, a low incidence of MCC and the lack of prospective control trials have made it unclear which modality of therapy offers the most benefit. In fact, two large retrospective National Cancer Database studies have made opposing conclusions with regard to the benefit of surgical lymphadenectomy over RT or vice versa [118,119]. It is common to recommend postoperative RT to the primary site due to a propensity for dermal metastases and local recurrence despite the presence of wide margins. Indications include positive or narrow margins or the presence of other risk factors, including lymphovascular invasion, or positive SLNB [105]. RT is also often recommended or considered for draining nodal basins in patients with obvious nodal disease, positive SLNB, a high risk of subclinical disease, or a high risk of false-negative SLNB. Even N0 patients with negative SLNB can be considered for RT given the aggressive nature of MCC [105]. One prospective trial was performed on this topic, randomizing patients with MCC to observation vs. regional RT. It showed no significant improvement in overall or progression-free survival, but decreased regional recurrence [120].

When surgical lymphadenectomy is performed for squamous cell carcinoma, the extent of neck dissection should be in line with the extent of nodal burden (i.e., clinically positive nodal disease should be treated more aggressively than clinically negative disease and those with many nodes or extracapsular extension should also be treated more aggressively, removing lymph node levels beyond those clinically involved) [105,106]. As one might expect, again, the exact extent of dissection is unknown for this rare tumor. As a result, one study applied a similar approach regarding the level of parotidectomy and neck dissection for cSCC to MCC, citing that it is at least as aggressive, if not more [121]. There was one specific study from the Moffit Cancer Center (Tampa, FL, USA), however, that found improved locoregional control and disease-specific survival in patients who underwent postoperative RT with pathologic or clinically positive lymph nodes, but not in patients with negative lymph nodes [122]. Postoperative chemotherapy or chemotherapy in conjunction with RT has shown mixed results. One study has shown a possible role for chemotherapy in the setting of high-risk MCC, specifically in those with positive margins or a large tumor size [123].

As displayed above, the management of the lymph node basin is a convoluted and difficult topic with a paucity of comparable data. As such, the NCCN has made the following recommendations based on clinical node-negative or node-positive disease [106]. For those with clinically node-negative disease and negative SLNB, observation can be considered and RT to the neck can be offered to those who are at high risk. Risk is a relative determination, but includes those with immunosuppression, those with an increased risk of false-negative SLNB, failure of SLNB, and a location in head and neck with variable lymph node drainage, or advanced-stage primary tumors [124]. For those with clinically node-negative disease and positive SLNB, the recommendation is to undergo neck dissection and/or RT. Decisions regarding RT to the primary site can be made distinctly from those related to the neck in the case of aggressive histologic indicators, advanced local disease, or other concerns. A summary of this topic is beyond the scope of this review, but it is actively being studied regarding survival benefit [125]. A brief comparative summary is shown in Table 3.

Neck management, in summary, involves the selection of appropriate levels of neck dissection based on the anatomic location of the primary cutaneous lesion. Adjuvant RT after surgical lymphadenectomy is recommended for those with multiple positive lymph nodes or extracapsular extension. For those in which SLNB was either not performed or failed, RT to the nodal bed should almost always be included. For those with biopsy-proven clinically node-positive disease, the recommendation is for lymph node dissection and/or primary RT. For high volume nodal disease, combined surgery and RT has generally been preferred, though this may change with recent advancements in immunotherapy. Adjuvant systemic therapy after lymph node dissection has variable recommendations but is currently generally only used in select cases such as stage IV disease or metastatic disease. Immunotherapy is currently being evaluated further as a novel adjuvant therapy for the intermediate stages of disease. Pembrolizumab, nivolumab, and avelumab are all active against MCC [126,127,128,129]. Whether they will eventually have a role in early treatment algorithms has yet to be determined, but adjuvant protocols for high-risk patients are in progress, and are promising in terms of early data [126,127,128,129].

### 2.5. Eccrine Cell Carcinoma

Eccrine cell carcinoma (EC) is a rare carcinoma that originates from the eccrine sweat glands of the skin and accounts for less than 0.01% of diagnosed cutaneous malignancies [130]. Eccrine carcinoma has a high rate of mortality and recurrence, its mortality exceeding that of melanoma [131]. It is subclassified into groups based on predominant cell origin: eccrine, apocrine, mixed, and unclassifiable. There are many subtypes, some of which include porocarcinoma, hidradenocarcinoma, malignant spiroadenocarcinoma, malignant cylindroma, syringoid eccrine carcinoma, microcystic adnexal carcinoma, mucinous carcinoma, adenoid cystic carcinoma, ductal papillary adenocarcinoma, eccrine ductal carcinoma, basaloid eccrine carcinoma, clear cell eccrine carcinoma, and non-specified sweat gland carcinoma [132]. Regarding metastasis to lymph nodes and beyond, prognosis is poor. Mortality has been quoted at 80% and with a 10-year survival rate as low as 9% [131]. Eccrine carcinomas occur throughout the body, among which 25% are found to occur in the head and neck [133].

With regard to pathology, ECC is classified into low grade and high grade [134]. Immunohistochemistry and serum cancer markers for these entities have also been variable [135,136]. Additionally, tumors with aggressive potential can appear much less aggressive on histological analysis [134]. When arriving at the histological diagnosis, however, one must ensure that cutaneous metastases from a visceral primary adenocarcinoma are excluded [134].

As may be expected of an already rare tumor, many subtypes further complicate the methodical study of the intricacy of each. Most data include case reports and all subsites of the body. As a result, there are no formal guidelines with regard to clinical presentation, appropriate workup, role for surgery, RT, and chemotherapy, and the management of nodal disease. There are a multitude of case reports, but this is obviously not sufficient to give summary recommendations. Historically, the standard of care is surgical resection with negative margins [137]. RT and chemotherapy have not proven successful [138]. Given its aggressive nature, SLNB was studied by Bogner et al. and showed positive results [139]. There were, however, only five patients in the study, only one of whom had a primary lesion in the head and neck. Other similarly small studies corroborated this method both in the head and neck and other subsites [135,140,141].

At this juncture, more research and analysis must be conducted to establish clinical care guidelines. Given its rarity and high mortality, management and treatment should be discussed with a multidisciplinary tumor board. For high-grade lesions with a significant depth of invasion and other aggressive features, nodal metastases are a risk, and it would appear reasonable to perform SLNB, but data are lacking.

### 2.6. Basal Cell Carcinoma

Basal cell carcinoma is the most common subtype of NMSC [20]. Fortunately, despite its increasing incidence, it is typically not prone to lymphatic or distant metastasis, but can be locally destructive [1]. BCC originates in the basal cell layer of the epidermis. Risk factors are similar to other NMSC, with ultraviolet exposure being one of the primary risk factors [4]. It is classified into different subtypes including nodular, superficial, and infiltrative, of which nodular makes up the majority [142]. After tissue diagnosis, imaging workup depends on the size and the number of risk factors present. CT allows for the assessment of deep margins and possible bony involvement.

While primary BCC is common, it is extremely rare for BCC to metastasize. Estimates have quoted the rate of metastasis to be between 0.003 percent and 0.5 percent of cases [143]. One hypothesis for extremely low rates of metastasis is that BCC almost always arises from hair-bearing skin and it requires the loose connective tissue present in the dermal stroma [4]. As this dermal tissue is not present elsewhere, it makes extradermal spread very difficult. Given this rarity in metastasis, there is no need for PET/CT or other imaging of the neck. Furthermore, once primary tumor resection has taken place, there is usually no indication for SLNB or a further evaluation of the lymph nodes. Although incredibly rare, lymphatic metastasis is possible and has been mentioned in case reports [143]. Thus, if clinical suspicion is present, further workup with neck imaging should be obtained. This has been mentioned particularly in case reports regarding rare cutaneous basosquamous carcinoma [144,145]. If clinical metastases to lymph nodes occur, then a standard dissection of the cervical lymphatics is needed based on the location of the primary [70], just as one might perform for squamous cell carcinoma. As mentioned, this is exceedingly rare for this histopathology.

### 2.7. Other Non-Melanoma Skin Cancers

Other subtypes of NMSC exist and include, but are not limited to, cutaneous lymphoma, dermatofibrosarcoma protuberans, Kaposi’s sarcoma, angiosarcoma, and other cutaneous sarcomas [146]. While there are some data surrounding these lesions, there is a paucity of data specifically regarding lymph node metastases and their management. In general, non-epithelial malignancies will not receive elective lymphadenectomy or SLNB, and surgery will focus on the primary site. There may be exceptions, underscoring the need for these tumors to always be treated in conjunction with a multidisciplinary tumor board.

## 3. Clinical Trials and New Treatment Options

Currently, there is one major clinical trial being conducted to further understand the role of SLNB in cutaneous SCC of the head and neck [147]. This will include T2-T4 patients with clinically and radiologically node-negative disease. All patients will undergo Mohs micrographic surgical resection followed by SLNB. This study will further aid our understanding of the necessity of SLNB. Given their rarity, these trials would be difficult or even impossible to run for tumors such as MCC or EC. While the primary focus of this review is lymph node metastasis of NMSC, there are exciting new treatment options and trials that have a high propensity to change management algorithms in the near future. A recent review by Cives et al. has outlined some of these developing options [148]. For further information, we suggest consulting www.clinicaltrials.gov (accessed on 13 August 2023).

## 4. Conclusions and Future Directions

Non-melanoma skin cancer is very common and represents a heterogeneous group of lesions, most of which can have excellent outcomes, particularly if treated in their early stages. Each subtype behaves very differently and thus requires a careful treatment regimen based on the clinical information available. There is well-established literature for the treatment of most primary lesions, particularly for more common lesions such as squamous cell carcinoma and basal cell carcinoma. When managing complex primary lesions and lymph node metastases, however, there remains a gap in nearly every subtype of NMSC. Rare tumors such as Merkel cell carcinoma and eccrine cell carcinoma have abundant research opportunities simply because of their rarity. SLNB is being developed as a major diagnostic modality for most NMSC, excluding BCC when clinically N0. Further research is needed to determine the optimal treatment regimen for occult nodal disease and whether or not SLNB should become the standard of care for certain high-risk tumors or high-risk patient populations. For clinically evident nodal disease, neck dissection and/or RT can be offered, depending on histology and stage. Neck dissections are favored for higher volume disease, and adjuvant RT is used when the final pathology indicates high risk. Immunotherapy is an emerging treatment modality that shows promising results. Further research is needed to better define the role it will have in the treatment algorithms in the future.

## Figures and Tables

**Table 1:** Comparison of staging classifications from the American Joint Committee on Cancer (AJCC) 8th Edition and Brigham and Women’s Hospital (BWH).

Tumor Staging System	AJCC 8th Edition	BWH
T1	<2 cm in greatest diameter	0 High-risk factors *
T2 ^Δ^	≥2 cm, but <4 cm in greatest diameter	N/A
T2a	N/A	1 High-risk factor
T2b	N/A	2–3 High-risk factors
T3 ^†^	Tumor ≥4 cm in greatest diameter or minor bone invasion or perineural invasion or deep invasion	4 High-risk factors or bone invasion
T4 ^Δ^		
T4a	Tumor with gross cortical bone and/or marrow invasion	N/A
T4b	Tumor with skull bone invasion and/or skull base foramen involvement	N/A

Abbreviations: AJCC—American Joint Committee on Cancer, BWH—Brigham and Women’s Hospital, N/A—Not Applicable. * BWH high-risk factors: ≥2 cm, poorly differentiated histology, perineural invasion of nerve(s) ≥0.1 mm in caliber, or tumor invasion beyond subcutaneous fat (excluding bone invasion, which upgrades tumor to BWH stage T3). ^†^ Deep invasion defined as invasion beyond the subcutaneous fat or >6 mm (as measured from the granular layer of adjacent normal epidermis to the base of the tumor), perineural invasion defined as tumor cells in the nerve sheath of a nerve lying deeper than the dermis or measuring 0.1 mm or larger in caliber or presenting with clinical or radiographic involvement of named nerves without skull base invasion or transgression. ^Δ^ There is no distinction between T2a and T2b in the AJCC 8th Edition staging system. There is no T4 distinction in the BWH Staging System.

**Table 2:** Comparison of key studies in establishing risk criteria for cutaneous squamous cell carcinoma and incidence of nodal metastasis.

Durham et al. [47]	Gore et al. [48]	Haisma et al. [53]
Study Inclusion Criteria for High-Risk Cutaneous Squamous Cell Carcinoma		
Locally recurrent	Local recurrence in the setting of adequate prior resection margins	N/A *
Occurrence in a prior radiation or chronic inflammation and/or ulcer site	Carcinoma in a pre-existing scar	
Perineural invasion	Perineural invasion	
Angiolymphatic invasion	Lymphovascular invasion	
Immunosuppression	Immunocompromise	
Size of 1 cm or more on cheek, forehead, scalp, neck, or 0.6 cm or more on face mask area	Tumor size > 2 cm	
Poorly differentiated	Poorly differentiated	
Clark level of IV or V	Invasion into subcutaneous fat or tumor thickness > 5 mm	
Breslow depth of 2 mm or more	Location on ear or lip	
Rapid growth		
**Risk criteria identified after sentinel lymph node biopsy or development of neck metastasis**		
Perineural invasion	Perineural invasion	Ear location
Angiolymphatic invasion	Lymphovascular invasion	Moderate or poor differentiation
Clinical size	Number of high-risk tumor factors	Tumor diameter > 50 mm
		Tumor thickness > 2 mm
**Incidence of nodal metastasis**		
15%	14%	16%

Abbreviations: N/A—Not applicable. [47,48,53]. * This study looked at a large cohort of patients without delineating between high-risk patient or tumor factors before analysis. It was included, however, because it involved a large patient cohort and is thus better powered to identify risk criteria.

**Table 3:** Summary of standard neck treatment for non-melanoma skin cancer.

**Squamous Cell Carcinoma [6,7,8,9,10,11,12,13,14,15,16,17,18,19,20,21,22,23,24,25,26,27,28,29,30,31,32,33,34,35,36,37,38,39,40,41,42,43,44,45,46,47,48,49,50,51,52,53,54,55,56,57,58,59,60,61,62,63,64,65,66,67,68,69,70,71,72,73,74,75,76,77,78,79,80,81,82,83]**			
**T1-T2N0**	**T3N0**	**T4N0**	**N+**
Primary resection, consider SLNB for high risk	Primary resection, consider SLNB vs elective neck dissection (END) +/− parotid vs. RT to nodal basin	T4 lesions may not be amenable to injection that fully encompasses lesion. END vs RT to nodal basin. Consider induction immunotherapy as an alternative to radical surgery.	Therapeutic lymphadenectomy (neck and/or parotid as indicated) in conjunction with primary resection. Adjuvant therapy based on final pathology report
**Merkel Cell Carcinoma [84,85,86,87,88,89,90,91,92,93,94,95,96,97,98,99,100,101,102,103,104,105,106,107,108,109,110,111,112,113,114,115,116,117,118,119,120,121,122,123,124,125,126,127,128,129]**			
**T1-T2N0**	**T3N0**	**T4N0**	**N+**
Resect primary, SLNB recommended	Resect primary, consider SLNB vs END +/− parotid vs. RT to nodal basin	If surgery is performed, consider END. SLNB may be an option if the lesion is not too large and invasive to fully inject. Consider immunotherapy upfront due to high rate of distant disease.	Therapeutic lymphadenectomy neck +/− parotid. If massive resection is necessary, including extensive adenopathy, consider induction immunotherapy (high rate of distant metastases)
**Eccrine Cell Carcinoma [130,131,132,133,134,135,136,137,138,139,140,141]**			
**T1-T2N0**	**T3N0**	**T4N0**	**N+**
Resect primary tumor. Very limited data available on SLNB which seems logical.	Resect primary tumor. Very limited data available on SLNB vs. END, either of which could be argued for.	Resect primary tumor. Very limited data available on SLNB vs. END, either of which could be argued for. Adjuvant radiation may be necessary.	Therapeutic lymphadenectomy, neck +/− parotid, with primary resection. Radiation probably will be indicated.
**Basal Cell Carcinoma [142,143,144,145,146]**			
**T1-T2N0**	**T3N0**	**T4N0**	**N+**
Primary resection only	Primary resection only	Primary resection only	Therapeutic Lymphadenectomy
**Non-epithelial Malignancies**			
No surgical management of lymphatics, even if primary is managed surgically.			Management would depend on histology, but may require therapeutic lymphadenectomy

## Data Availability

The data presented in this study are available in this article.

## References

[1] Kansara S., Bell D., Weber R. (2020). Surgical management of non melanoma skin cancer of the head and neck. Oral Oncol..

[2] Newlands C., Currie R., Memon A., Whitaker S., Woolford T. (2016). Non-melanoma skin cancer: United Kingdom National Multidisciplinary Guidelines. J. Laryngol. Otol..

[3] Cecchi R., Buralli L., De Gaudioc C. (2006). Sentinel lymphonodectomy in non-melanoma skin cancers. Chir. Ital..

[4] Flint P. (2015). Cummings Otolaryngology Head and Neck Surgery.

[5] Rudolph C., Schnoor M., Eisemann N., Katalinic A. (2015). Incidence trends of nonmelanoma skin cancer in Germany from 1998 to 2010. J. Dtsch. Dermatol. Ges. J. Ger. Soc. Dermatol. JDDG.

[6] Schmults C.D., Karia P.S., Carter J.B., Han J., Qureshi A.A. (2013). Factors predictive of recurrence and death from cutaneous squamous cell carcinoma: A 10-year, single-institution cohort study. JAMA Dermatol..

[7] Varra V., Woody N.M., Reddy C., Joshi N.P., Geiger J., Adelstein D.J., Burkey B.B., Scharpf J., Prendes B., Lamarre E.D. (2018). Suboptimal Outcomes in Cutaneous Squamous Cell Cancer of the Head and Neck with Nodal Metastases. Anticancer Res..

[8] Rogers H.W., Weinstock M.A., Feldman S.R., Coldiron B.M. (2015). Incidence Estimate of Nonmelanoma Skin Cancer (Keratinocyte Carcinomas) in the U.S. Population, 2012. JAMA Dermatol..

[9] Wehner M.R., Shive M.L., Chren M.-M., Han J., Qureshi A.A., Linos E. (2012). Indoor tanning and non-melanoma skin cancer: Systematic review and meta-analysis. BMJ.

[10] Ramsay H.M., Fryer A.A., Hawley C.M., Smith A.G., Nicol D.L., Harden P.N. (2003). Factors associated with nonmelanoma skin cancer following renal transplantation in Queensland, Australia. J. Am. Acad. Dermatol..

[11] Senet P., Combemale P., Debure C., Baudot N., Machet L., Aout M., Vicaut E., Lok C. (2012). Angio-Dermatology Group of The French Society of Dermatology Malignancy and chronic leg ulcers: The value of systematic wound biopsies: A prospective, multicenter, cross-sectional study. Arch. Dermatol..

[12] Kromberg J.G., Castle D., Zwane E.M., Jenkins T. (1989). Albinism and skin cancer in Southern Africa. Clin. Genet..

[13] Thielmann H.W., Popanda O., Edler L., Jung E.G. (1991). Clinical symptoms and DNA repair characteristics of xeroderma pigmentosum patients from Germany. Cancer Res..

[14] Miranda M.B., Lauseker M., Kraus M.-P., Proetel U., Hanfstein B., Fabarius A., Baerlocher G.M., Heim D., Hossfeld D.K., Kolb H.-J. (2016). Secondary malignancies in chronic myeloid leukemia patients after imatinib-based treatment: Long-term observation in CML Study IV. Leukemia.

[15] Que S.K.T., Zwald F.O., Schmults C.D. (2018). Cutaneous squamous cell carcinoma: Incidence, risk factors, diagnosis, and staging. J. Am. Acad. Dermatol..

[16] Kallini J.R., Hamed N., Khachemoune A. (2015). Squamous cell carcinoma of the skin: Epidemiology, classification, management, and novel trends. Int. J. Dermatol..

[17] Ciążyńska M., Kamińska-Winciorek G., Lange D., Lewandowski B., Reich A., Sławińska M., Pabianek M., Szczepaniak K., Hankiewicz A., Ułańska M. (2021). The incidence and clinical analysis of non-melanoma skin cancer. Sci. Rep..

[18] Brougham N.D.L., Tan S.T. (2014). The incidence and risk factors of metastasis for cutaneous squamous cell carcinoma--implications on the T-classification system. J. Surg. Oncol..

[19] Leiter U., Keim U., Eigentler T., Katalinic A., Holleczek B., Martus P., Garbe C. (2017). Incidence, Mortality, and Trends of Nonmelanoma Skin Cancer in Germany. J. Investig. Dermatol..

[20] Miller D.L., Weinstock M.A. (1994). Nonmelanoma skin cancer in the United States: Incidence. J. Am. Acad. Dermatol..

[21] Alam M., Ratner D. (2001). Cutaneous squamous-cell carcinoma. N. Engl. J. Med..

[22] Wilkie M.D., Lancaster J., Roland N.J., Jones T.M. (2021). Elective management of regional nodal basins in cutaneous squamous cell carcinoma of the head and neck: Controversies and contemporary perspectives. Oral Oncol..

[23] Silverman M.K., Kopf A.W., Grin C.M., Bart R.S., Levenstein M.J. (1991). Recurrence rates of treated basal cell carcinomas. Part 1: Overview. J. Dermatol. Surg. Oncol..

[24] Silverman M.K., Kopf A.W., Grin C.M., Bart R.S., Levenstein M.J. (1991). Recurrence rates of treated basal cell carcinomas. Part 2: Curettage-electrodesiccation. J. Dermatol. Surg. Oncol..

[25] Schmults C.D. (2021). NCCN Clinical Practice Guidelines in Oncology. Squamous Cell Skin Cancer. J. Natl. Compr. Canc. Netw..

[26] Rowe D.E., Carroll R.J., Day C.L. (1992). Prognostic factors for local recurrence, metastasis, and survival rates in squamous cell carcinoma of the skin, ear, and lip. Implications for treatment modality selection. J. Am. Acad. Dermatol..

[27] Thompson A.K., Kelley B.F., Prokop L.J., Murad M.H., Baum C.L. (2016). Risk Factors for Cutaneous Squamous Cell Carcinoma Recurrence, Metastasis, and Disease-Specific Death: A Systematic Review and Meta-analysis. JAMA Dermatol..

[28] Kyrgidis A., Tzellos T.G., Kechagias N., Patrikidou A., Xirou P., Kitikidou K., Bourlidou E., Vahtsevanos K., Antoniades K. (2010). Cutaneous squamous cell carcinoma (SCC) of the head and neck: Risk factors of overall and recurrence-free survival. Eur. J. Cancer.

[29] Brantsch K.D., Meisner C., Schönfisch B., Trilling B., Wehner-Caroli J., Röcken M., Breuninger H. (2008). Analysis of risk factors determining prognosis of cutaneous squamous-cell carcinoma: A prospective study. Lancet Oncol..

[30] Gonzalez-Guerrero M., Martínez-Camblor P., Vivanco B., Fernández-Vega I., Munguía-Calzada P., Gonzalez-Gutierrez M.P., Rodrigo J.P., Galache C., Santos-Juanes J. (2017). The adverse prognostic effect of tumor budding on the evolution of cutaneous head and neck squamous cell carcinoma. J. Am. Acad. Dermatol..

[31] Sahovaler A., Krishnan R.J., Yeh D.H., Zhou Q., Palma D., Fung K., Yoo J., Nichols A., MacNeil S.D. (2019). Outcomes of Cutaneous Squamous Cell Carcinoma in the Head and Neck Region with Regional Lymph Node Metastasis: A Systematic Review and Meta-analysis. JAMA Otolaryngol. Head Neck Surg..

[32] Rangwala S., Tsai K.Y. (2011). Roles of the immune system in skin cancer. Br. J. Dermatol..

[33] Yu S.H., Bordeaux J.S., Baron E.D. (2014). The immune system and skin cancer. Adv. Exp. Med. Biol..

[34] Santos-Juanes J., Fernández-Vega I., Lorenzo-Herrero S., Sordo-Bahamonde C., Martínez-Camblor P., García-Pedrero J.M., Vivanco B., Galache-Osuna C., Vazquez-Lopez F., Gonzalez S. (2019). Lectin-like transcript 1 (LLT1) expression is associated with nodal metastasis in patients with head and neck cutaneous squamous cell carcinoma. Arch. Dermatol. Res..

[35] Munguía-Calzada P., Fernández-Vega I., Martínez-Camblor P., Díaz-Coto S., García-Pedrero J.M., Vivanco B., Osuna C.G., Vazquez-Lopez F., Rodrigo J.P., Santos-Juanes J. (2019). Correlation of focal adhesion kinase expression with nodal metastasis in patients with head and neck cutaneous squamous cell carcinoma. Head Neck.

[36] García-Pedrero J.M., Martínez-Camblor P., Diaz-Coto S., Munguia-Calzada P., Vallina-Alvarez A., Vazquez-Lopez F., Rodrigo J.P., Santos-Juanes J. (2017). Tumor programmed cell death ligand 1 expression correlates with nodal metastasis in patients with cutaneous squamous cell carcinoma of the head and neck. J. Am. Acad. Dermatol..

[37] Burton K.A., Ashack K.A., Khachemoune A. (2016). Cutaneous Squamous Cell Carcinoma: A Review of High-Risk and Metastatic Disease. Am. J. Clin. Dermatol..

[38] Mooney C.P., Martin R.C.W., Dirven R., Ashford B.G., Shannon K., Palme C.E., Ngo Q., Wykes J., Davies S., Gao K. (2019). Sentinel Node Biopsy in 105 High-Risk Cutaneous SCCs of the Head and Neck: Results of a Multicenter Prospective Study. Ann. Surg. Oncol..

[39] Weinberg A.S., Ogle C.A., Shim E.K. (2007). Metastatic Cutaneous Squamous Cell Carcinoma: An Update. Dermatol. Surg..

[40] Venables Z.C., Autier P., Nijsten T., Wong K.F., Langan S.M., Rous B., Broggio J., Harwood C., Henson K., Proby C.M. (2019). Nationwide Incidence of Metastatic Cutaneous Squamous Cell Carcinoma in England. JAMA Dermatol..

[41] Epstein E., Epstein N.N., Bragg K., Linden G. (1968). Metastases from squamous cell carcinomas of the skin. Arch. Dermatol..

[42] Peat B., Insull P., Ayers R. (2012). Risk stratification for metastasis from cutaneous squamous cell carcinoma of the head and neck. ANZ J. Surg..

[43] Wermker K., Kluwig J., Schipmann S., Klein M., Schulze H.J., Hallermann C. (2015). Prediction score for lymph node metastasis from cutaneous squamous cell carcinoma of the external ear. Eur. J. Surg. Oncol..

[44] Williams L.S., Mancuso A.A., Mendenhall W.M. (2001). Perineural spread of cutaneous squamous and basal cell carcinoma: CT and MR detection and its impact on patient management and prognosis. Int. J. Radiat. Oncol. Biol. Phys..

[45] de Bondt R.B.J., Nelemans P.J., Hofman P.A.M., Casselman J.W., Kremer B., van Engelshoven J.M.A., Beets-Tan R.G.H. (2007). Detection of lymph node metastases in head and neck cancer: A meta-analysis comparing US, USgFNAC, CT and MR imaging. Eur. J. Radiol..

[46] Nouri K., Rivas M.P., Pedroso F., Bhatia R., Civantos F. (2004). Sentinel lymph node biopsy for high-risk cutaneous squamous cell carcinoma of the head and neck. Arch. Dermatol..

[47] Durham A.B., Lowe L., Malloy K.M., McHugh J.B., Bradford C.R., Chubb H., Johnson T.M., McLean S.A. (2016). Sentinel Lymph Node Biopsy for Cutaneous Squamous Cell Carcinoma on the Head and Neck. JAMA Otolaryngol. Head Neck Surg..

[48] Gore S.M., Shaw D., Martin R.C.W., Kelder W., Roth K., Uren R., Gao K., Davies S., Ashford B.G., Ngo Q. (2016). Prospective study of sentinel node biopsy for high-risk cutaneous squamous cell carcinoma of the head and neck. Head Neck.

[49] Ross A.S., Schmults C.D. (2006). Sentinel lymph node biopsy in cutaneous squamous cell carcinoma: A systematic review of the English literature. Dermatol. Surg..

[50] Kwon S., Dong Z.M., Wu P.C. (2011). Sentinel lymph node biopsy for high-risk cutaneous squamous cell carcinoma: Clinical experience and review of literature. World J. Surg. Oncol..

[51] Takahashi A., Imafuku S., Nakayama J., Nakaura J., Ito K., Shibayama Y. (2014). Sentinel node biopsy for high-risk cutaneous squamous cell carcinoma. Eur. J. Surg. Oncol..

[52] Lubov J., Labbé M., Sioufi K., Morand G.B., Hier M.P., Khanna M., Sultanem K., Mlynarek A.M. (2021). Prognostic factors of head and neck cutaneous squamous cell carcinoma: A systematic review. J. Otolaryngol. Head Neck Surg..

[53] Haisma M.S., Plaat B.E.C., Bijl H.P., Roodenburg J.L.N., Diercks G.F.H., Romeijn T.R., Terra J.B. (2016). Multivariate analysis of potential risk factors for lymph node metastasis in patients with cutaneous squamous cell carcinoma of the head and neck. J. Am. Acad. Dermatol..

[54] Soleymani T., Brodland D.G., Arzeno J., Sharon D.J., Zitelli J.A. (2023). Clinical outcomes of high-risk cutaneous squamous cell carcinomas treated with Mohs surgery alone: An analysis of local recurrence, regional nodal metastases, progression-free survival, and disease-specific death. J. Am. Acad. Dermatol..

[55] Cancer Staging Form Supplement American College of Surgeons. http://www.facs.org/quality-programs/cancer/ajcc/cancer-staging/form-supplement.

[56] Jambusaria-Pahlajani A., Kanetsky P.A., Karia P.S., Hwang W.-T., Gelfand J.M., Whalen F.M., Elenitsas R., Xu X., Schmults C.D. (2013). Evaluation of AJCC tumor staging for cutaneous squamous cell carcinoma and a proposed alternative tumor staging system. JAMA Dermatol..

[57] Ruiz E.S., Karia P.S., Besaw R., Schmults C.D. (2019). Performance of the American Joint Committee on Cancer Staging Manual, 8th Edition vs the Brigham and Women’s Hospital Tumor Classification System for Cutaneous Squamous Cell Carcinoma. JAMA Dermatol..

[58] Xiao Y., Yuan S., Liu F., Liu B., Zhu J., He W., Li W., Kan Q. (2018). Comparison between wait-and-see policy and elective neck dissection in clinically N0 cutaneous squamous cell carcinoma of head and neck. Medicine.

[59] Cannon R.B., Dundar Y., Thomas A., Monroe M.M., Buchmann L.O., Witt B.L., Sowder A.M., Hunt J.P. (2017). Elective Neck Dissection for Head and Neck Cutaneous Squamous Cell Carcinoma with Skull Base Invasion. Otolaryngol. Head Neck Surg..

[60] Amit M., Liu C., Mansour J., Gleber-Netto F.O., Tam S., Baruch E.N., Aashiq M., El-Naggar A.K., Moreno A.C., Rosenthal D.I. (2021). Elective neck dissection versus observation in patients with head and neck cutaneous squamous cell carcinoma. Cancer.

[61] Southwell K.E., Chaplin J.M., Eisenberg R.L., McIvor N.P., Morton R.P. (2006). Effect of immunocompromise on metastatic cutaneous squamous cell carcinoma in the parotid and neck. Head Neck.

[62] Palme C.E., O’Brien C.J., Veness M.J., McNeil E.B., Bron L.P., Morgan G.J. (2003). Extent of parotid disease influences outcome in patients with metastatic cutaneous squamous cell carcinoma. Arch. Otolaryngol. Head Neck Surg..

[63] Veness M.J., Palme C.E., Smith M., Cakir B., Morgan G.J., Kalnins I. (2003). Cutaneous head and neck squamous cell carcinoma metastatic to cervical lymph nodes (nonparotid): A better outcome with surgery and adjuvant radiotherapy. Laryngoscope.

[64] Gurney B., Newlands C. (2014). Management of regional metastatic disease in head and neck cutaneous malignancy. 1. Cutaneous squamous cell carcinoma. Br. J. Oral Maxillofac. Surg..

[65] O’Brien C.J., McNeil E.B., McMahon J.D., Pathak I., Lauer C.S., Jackson M.A. (2002). Significance of clinical stage, extent of surgery, and pathologic findings in metastatic cutaneous squamous carcinoma of the parotid gland. Head Neck.

[66] Gurudutt V.V., Genden E.M. (2011). Cutaneous squamous cell carcinoma of the head and neck. J. Skin Cancer.

[67] Agnese D.M., Maupin R., Tillman B., Pozderac R.D., Magro C., Walker M.J. (2007). Head and neck melanoma in the sentinel lymph node era. Arch. Otolaryngol. Head Neck Surg..

[68] Rotman A., Kerr S.J., Giddings C.E.B. (2019). Elective neck dissection in metastatic cutaneous squamous cell carcinoma to the parotid gland: A systematic review and meta-analysis. Head Neck.

[69] Vauterin T.J., Veness M.J., Morgan G.J., Poulsen M.G., O’Brien C.J. (2006). Patterns of lymph node spread of cutaneous squamous cell carcinoma of the head and neck. Head Neck.

[70] Ebrahimi A., Moncrieff M.D., Clark J.R., Shannon K.F., Gao K., Milross C.G., O’Brien C.J. (2010). Predicting the pattern of regional metastases from cutaneous squamous cell carcinoma of the head and neck based on location of the primary. Head Neck.

[71] D’Souza J., Clark J. (2011). Management of the neck in metastatic cutaneous squamous cell carcinoma of the head and neck. Curr. Opin. Otolaryngol. Head Neck Surg..

[72] Mierzwa M.L. (2019). Radiotherapy for Skin Cancers of the Face, Head, and Neck. Facial Plast. Surg. Clin..

[73] Veness M.J., Morgan G.J., Palme C.E., Gebski V. (2005). Surgery and adjuvant radiotherapy in patients with cutaneous head and neck squamous cell carcinoma metastatic to lymph nodes: Combined treatment should be considered best practice. Laryngoscope.

[74] Peiffer N., Kutz J.W., Myers L.L., Isaacson B., Sumer B.D., Truelson J.M., Ahn C., Roland P.S. (2011). Patterns of regional metastasis in advanced stage cutaneous squamous cell carcinoma of the auricle. Otolaryngol. Head Neck Surg..

[75] Wang J.T., Palme C.E., Morgan G.J., Gebski V., Wang A.Y., Veness M.J. (2012). Predictors of outcome in patients with metastatic cutaneous head and neck squamous cell carcinoma involving cervical lymph nodes: Improved survival with the addition of adjuvant radiotherapy. Head Neck.

[76] Ruiz E.S., Kus K.J.B., Smile T.D., Murad F., Zhou G., Ilori E.O., Schoenfeld J.D., Margalit D.N., Tishler R.B., Vidimos A.T. (2022). Adjuvant radiation following clear margin resection of high T-stage cutaneous squamous cell carcinoma halves the risk of local and locoregional recurrence: A dual-center retrospective study. J. Am. Acad. Dermatol..

[77] Schmidt C., Martin J.M., Khoo E., Plank A., Grigg R. (2015). Outcomes of nodal metastatic cutaneous squamous cell carcinoma of the head and neck treated in a regional center. Head Neck.

[78] Forest V.-I., Clark J.J., Veness M.J., Milross C. (2010). N1S3: A revised staging system for head and neck cutaneous squamous cell carcinoma with lymph node metastases: Results of 2 Australian Cancer Centers. Cancer.

[79] Porceddu S.V., Bressel M., Poulsen M.G., Stoneley A., Veness M.J., Kenny L.M., Wratten C., Corry J., Cooper S., Fogarty G.B. (2018). Postoperative Concurrent Chemoradiotherapy Versus Postoperative Radiotherapy in High-Risk Cutaneous Squamous Cell Carcinoma of the Head and Neck: The Randomized Phase III TROG 05.01 Trial. J. Clin. Oncol..

[80] Migden M.R., Rischin D., Schmults C.D., Guminski A., Hauschild A., Lewis K.D., Chung C.H., Hernandez-Aya L., Lim A.M., Chang A.L.S. (2018). PD-1 Blockade with Cemiplimab in Advanced Cutaneous Squamous-Cell Carcinoma. N. Engl. J. Med..

[81] Ferrarotto R., Amit M., Nagarajan P., Rubin M.L., Yuan Y., Bell D., El-Naggar A.K., Johnson J.M., Morrison W.H., Rosenthal D.I. (2021). Pilot Phase II Trial of Neoadjuvant Immunotherapy in Locoregionally Advanced, Resectable Cutaneous Squamous Cell Carcinoma of the Head and Neck. Clin. Cancer Res..

[82] Ibrahim S.F., Kasprzak J.M., Hall M.A., Fitzgerald A.L., Siegel J.J., Kurley S.J., Covington K.R., Goldberg M.S., Farberg A.S., Trotter S.C. (2022). Enhanced metastatic risk assessment in cutaneous squamous cell carcinoma with the 40-gene expression profile test. Future Oncol. Lond. Engl..

[83] Arron S.T., Blalock T.W., Guenther J.M., Hyams D.M., Ibrahim S.F., Koyfman S.A., Wysong A. (2021). Clinical Considerations for Integrating Gene Expression Profiling into Cutaneous Squamous Cell Carcinoma Management. J. Drugs Dermatol. JDD.

[84] Walsh N.M., Cerroni L. (2021). Merkel cell carcinoma: A review. J. Cutan. Pathol..

[85] Paulson K.G., Park S.Y., Vandeven N.A., Lachance K., Thomas H., Chapuis A.G., Harms K.L., Thompson J.A., Bhatia S., Stang A. (2018). Merkel cell carcinoma: Current US incidence and projected increases based on changing demographics. J. Am. Acad. Dermatol..

[86] DeCaprio J.A. (2021). Molecular Pathogenesis of Merkel Cell Carcinoma. Annu. Rev. Pathol..

[87] Feng H., Shuda M., Chang Y., Moore P.S. (2008). Clonal integration of a polyomavirus in human Merkel cell carcinoma. Science.

[88] Moshiri A.S., Doumani R., Yelistratova L., Blom A., Lachance K., Shinohara M.M., Delaney M., Chang O., McArdle S., Thomas H. (2017). Polyomavirus-Negative Merkel Cell Carcinoma: A More Aggressive Subtype Based on Analysis of 282 Cases Using Multimodal Tumor Virus Detection. J. Investig. Dermatol..

[89] Harms P.W., Collie A.M.B., Hovelson D.H., Cani A.K., Verhaegen M.E., Patel R.M., Fullen D.R., Omata K., Dlugosz A.A., Tomlins S.A. (2016). Next generation sequencing of Cytokeratin 20-negative Merkel cell carcinoma reveals ultraviolet-signature mutations and recurrent TP53 and RB1 inactivation. Mod. Pathol..

[90] Heath M., Jaimes N., Lemos B., Mostaghimi A., Wang L.C., Peñas P.F., Nghiem P. (2008). Clinical characteristics of Merkel cell carcinoma at diagnosis in 195 patients: The AEIOU features. J. Am. Acad. Dermatol..

[91] Tarantola T.I., Vallow L.A., Halyard M.Y., Weenig R.H., Warschaw K.E., Grotz T.E., Jakub J.W., Roenigk R.K., Brewer J.D., Weaver A.L. (2013). Prognostic factors in Merkel cell carcinoma: Analysis of 240 cases. J. Am. Acad. Dermatol..

[92] Mazziotta C., Cervellera C.F., Lanzillotti C., Touzé A., Gaboriaud P., Tognon M., Martini F., Rotondo J.C. (2023). MicroRNA dysregulations in Merkel cell carcinoma: Molecular mechanisms and clinical applications. J. Med. Virol..

[93] Smith V.A., MaDan O.P., Lentsch E.J. (2012). Tumor location is an independent prognostic factor in head and neck Merkel cell carcinoma. Otolaryngol. Head Neck Surg..

[94] Lemos B.D., Storer B.E., Iyer J.G., Phillips J.L., Bichakjian C.K., Fang L.C., Johnson T.M., Liegeois-Kwon N.J., Otley C.C., Paulson K.G. (2010). Pathologic nodal evaluation improves prognostic accuracy in Merkel cell carcinoma: Analysis of 5823 cases as the basis of the first consensus staging system. J. Am. Acad. Dermatol..

[95] van Veenendaal L.M., van Akkooi A.C.J., Verhoef C., Grünhagen D.J., Klop W.M.C., Valk G.D., Tesselaar M.E.T. (2018). Merkel cell carcinoma: Clinical outcome and prognostic factors in 351 patients. J. Surg. Oncol..

[96] Smith F.O., Yue B., Marzban S.S., Walls B.L., Carr M., Jackson R.S., Puleo C.A., Padhya T., Cruse C.W., Gonzalez R.J. (2015). Both tumor depth and diameter are predictive of sentinel lymph node status and survival in Merkel cell carcinoma. Cancer.

[97] Fields R.C., Busam K.J., Chou J.F., Panageas K.S., Pulitzer M.P., Kraus D.H., Brady M.S., Coit D.G. (2011). Recurrence and survival in patients undergoing sentinel lymph node biopsy for merkel cell carcinoma: Analysis of 153 patients from a single institution. Ann. Surg. Oncol..

[98] Schwartz J.L., Griffith K.A., Lowe L., Wong S.L., McLean S.A., Fullen D.R., Lao C.D., Hayman J.A., Bradford C.R., Rees R.S. (2011). Features predicting sentinel lymph node positivity in Merkel cell carcinoma. J. Clin. Oncol..

[99] Feldmeyer L., Hudgens C.W., Ray-Lyons G., Nagarajan P., Aung P.P., Curry J.L., Torres-Cabala C.A., Mino B., Rodriguez-Canales J., Reuben A. (2016). Density, Distribution, and Composition of Immune Infiltrates Correlate with Survival in Merkel Cell Carcinoma. Clin. Cancer Res..

[100] Pellitteri P.K., Takes R.P., Lewis J.S., Devaney K.O., Harlor E.J., Strojan P., Rodrigo J.P., Suárez C., Rinaldo A., Medina J.E. (2012). Merkel cell carcinoma of the head and neck. Head Neck.

[101] Gupta S.G., Wang L.C., Peñas P.F., Gellenthin M., Lee S.J., Nghiem P. (2006). Sentinel lymph node biopsy for evaluation and treatment of patients with Merkel cell carcinoma: The Dana-Farber experience and meta-analysis of the literature. Arch. Dermatol..

[102] Colgan M.B., Tarantola T.I., Weaver A.L., Wiseman G.A., Roenigk R.K., Brewer J.D., Otley C.C. (2012). The predictive value of imaging studies in evaluating regional lymph node involvement in Merkel cell carcinoma. J. Am. Acad. Dermatol..

[103] Liu J., Larcos G., Howle J., Veness M. (2017). Lack of clinical impact of 18 F-fluorodeoxyglucose positron emission tomography with simultaneous computed tomography for stage I and II Merkel cell carcinoma with concurrent sentinel lymph node biopsy staging: A single institutional experience from Westmead Hospital, Sydney. Australas. J. Dermatol..

[104] Hawryluk E.B., O’Regan K.N., Sheehy N., Guo Y., Dorosario A., Sakellis C.G., Jacene H.A., Wang L.C. (2013). Positron emission tomography/computed tomography imaging in Merkel cell carcinoma: A study of 270 scans in 97 patients at the Dana-Farber/Brigham and Women’s Cancer Center. J. Am. Acad. Dermatol..

[105] NCCN Clinical Practice Guidelines in Oncology (2021). Merkel Cell Carcinoma. Version 1. https://www.nccn.org/professionals/physician_gls/pdf/mcc.pdf.

[106] Bichakjian C.K., Olencki T., Aasi S.Z., Alam M., Andersen J.S., Blitzblau R., Bowen G.M., Contreras C.M., Daniels G.A., Decker R. (2018). Merkel Cell Carcinoma, Version 1.2018, NCCN Clinical Practice Guidelines in On-cology. J. Natl. Compr. Cancer Netw. JNCCN.

[107] Horii A., Yoshida J., Honjo Y., Mitani K., Takashima S., Kubo T. (1998). Pre-operative assessment of metastatic parotid tumors. Auris. Nasus. Larynx.

[108] Kim H.J., Yoon D.Y., Hong J.H., Yun E.J., Baek S., Kim E.S., Park M.W., Kwon K.H. (2020). Intra-parotid lymph node metastasis in patients with non-cutaneous head and neck cancers: Clinical and imaging features for differentiation from simultaneous parotid primary tumor. Acta Radiol..

[109] Mehrany K., Otley C.C., Weenig R.H., Phillips P.K., Roenigk R.K., Nguyen T.H. (2002). A meta-analysis of the prognostic significance of sentinel lymph node status in Merkel cell carcinoma. Dermatol. Surg..

[110] Servy A., Maubec E., Sugier P.E., Grange F., Mansard S., Lesimple T., Marinho E., Couturaud B., Girod A., Albert S. (2016). Merkel cell carcinoma: Value of sentinel lymph-node status and adjuvant radiation therapy. Ann. Oncol..

[111] Kachare S.D., Wong J.H., Vohra N.A., Zervos E.E., Fitzgerald T.L. (2014). Sentinel lymph node biopsy is associated with improved survival in Merkel cell carcinoma. Ann. Surg. Oncol..

[112] Sims J.R., Grotz T.E., Pockaj B.A., Joseph R.W., Foote R.L., Otley C.C., Weaver A.L., Jakub J.W., Price D.L. (2018). Sentinel lymph node biopsy in Merkel cell carcinoma: The Mayo Clinic experience of 150 patients. Surg. Oncol..

[113] Fritsch V.A., Camp E.R., Lentsch E.J. (2014). Sentinel lymph node status in Merkel cell carcinoma of the head and neck: Not a predictor of survival. Head Neck.

[114] Asgari M.M., Sokil M.M., Warton E.M., Iyer J., Paulson K.G., Nghiem P. (2014). Effect of host, tumor, diagnostic, and treatment variables on outcomes in a large cohort with Merkel cell carcinoma. JAMA Dermatol..

[115] Liang E., Brower J.V., Rice S.R., Buehler D.G., Saha S., Kimple R.J. (2015). Merkel Cell Carcinoma Analysis of Outcomes: A 30-Year Experience. PLoS ONE.

[116] Righi A., Asioli S., Caliendo V., Macripò G., Picciotto F., Risio M., Eusebi V., Bussolati G. (2013). An ultrasonography-cytology protocol for the diagnostic management of regional nodes in a subset of patients with Merkel cell carcinoma of the skin. Br. J. Dermatol..

[117] Jabbour J., Cumming R., Scolyer R.A., Hruby G., Thompson J.F., Lee S. (2007). Merkel cell carcinoma: Assessing the effect of wide local excision, lymph node dissection, and radiotherapy on recurrence and survival in early-stage disease--results from a review of 82 consecutive cases diagnosed between 1992 and 2004. Ann. Surg. Oncol..

[118] Wright G.P., Holtzman M.P. (2018). Surgical resection improves median overall survival with marginal improvement in long-term survival when compared with definitive radiotherapy in Merkel cell carcinoma: A propensity score matched analysis of the National Cancer Database. Am. J. Surg..

[119] Adjuvant Radiation Therapy and Chemotherapy in Merkel Cell Carcinoma: Survival Analyses of 6908 Cases From the National Cancer Data Base—PubMed. https://pubmed.ncbi.nlm.nih.gov/27245173/.

[120] Jouary T., Leyral C., Dreno B., Doussau A., Sassolas B., Beylot-Barry M., Renaud-Vilmer C., Guillot B., Bernard P., Lok C. (2012). Adjuvant prophylactic regional radiotherapy versus observation in stage I Merkel cell carcinoma: A multicentric prospective randomized study. Ann. Oncol..

[121] Bowe C.M., Gurney B., Whitaker S., Newlands C. (2019). Management of regional metastatic disease in cutaneous malignancy of the head and neck. 3. Merkel cell carcinoma. Br. J. Oral Maxillofac. Surg..

[122] Strom T., Carr M., Zager J.S., Naghavi A., Smith F.O., Cruse C.W., Messina J.L., Russell J., Rao N.G., Fulp W. (2016). Radiation Therapy is Associated with Improved Outcomes in Merkel Cell Carcinoma. Ann. Surg. Oncol..

[123] Chen M.M., Roman S.A., Sosa J.A., Judson B.L. (2015). The role of adjuvant therapy in the management of head and neck merkel cell carcinoma: An analysis of 4815 patients. JAMA Otolaryngol. Head Neck Surg..

[124] Shnayder Y., Weed D.T., Arnold D.J., Gomez-Fernandez C., Bared A., Goodwin W.J., Civantos F.J. (2008). Management of the neck in Merkel cell carcinoma of the head and neck: University of Miami experience. Head Neck.

[125] Wong W.G., Stahl K., Olecki E.J., Holguin R.P., Pameijer C., Shen C. (2021). Survival Benefit of Guideline-Concordant Postoperative Radiation for Local Merkel Cell Carcinoma. J. Surg. Res..

[126] Nghiem P., Bhatia S., Lipson E.J., Sharfman W.H., Kudchadkar R.R., Brohl A.S., Friedlander P.A., Daud A., Kluger H.M., Reddy S.A. (2019). Durable Tumor Regression and Overall Survival in Patients with Advanced Merkel Cell Carcinoma Receiving Pembrolizumab as First-Line Therapy. J. Clin. Oncol..

[127] Topalian S.L., Bhatia S., Amin A., Kudchadkar R.R., Sharfman W.H., Lebbé C., Delord J.-P., Dunn L.A., Shinohara M.M., Kulikauskas R. (2020). Neoadjuvant Nivolumab for Patients with Resectable Merkel Cell Carcinoma in the CheckMate 358 Trial. J. Clin. Oncol..

[128] D’Angelo S.P., Bhatia S., Brohl A.S., Hamid O., Mehnert J.M., Terheyden P., Shih K.C., Brownell I., Lebbé C., Lewis K.D. (2020). Avelumab in patients with previously treated metastatic Merkel cell carcinoma: Long-term data and biomarker analyses from the single-arm phase 2 JAVELIN Merkel 200 trial. J. Immunother. Cancer.

[129] Colunga A., Pulliam T., Nghiem P. (2018). Merkel Cell Carcinoma in the Age of Immunotherapy: Facts and Hopes. Clin. Cancer Res..

[130] Sidiropoulos M., Sade S., Al-Habeeb A., Ghazarian D. (2011). Syringoid eccrine carcinoma: A clinicopathological and immunohistochemical study of four cases. J. Clin. Pathol..

[131] Larson K., Babiker H.M., Kovoor A., Liau J., Eldersveld J., Elquza E. (2018). Oral Capecitabine Achieves Response in Metastatic Eccrine Carcinoma. Case Rep. Oncol. Med..

[132] Kaseb H., Babiker H.M. (2021). Eccrine Carcinoma. StatPearls.

[133] Salih A.M., Kakamad F.H., Essa R.A., Rauf G.M., Masrur S.A., Shvan H.M., Rawezh Q.S., Hunar A.H., Dahat A.H., Othman S. (2016). Porocarcinoma: A systematic review of literature with a single case report. Int. J. Surg. Case Rep..

[134] van der Horst M.P.J., Brenn T. (2017). Update on Malignant Sweat Gland Tumors. Surg. Pathol. Clin..

[135] Gómez-Zubiaur A., Medina-Montalvo S., Vélez-Velázquez M.D., Polo-Rodríguez I. (2017). Eccrine Porocarcinoma: Patient Characteristics, Clinical and Histopathologic Features, and Treatment in 7 Cases. Actas Dermosifiliogr..

[136] Sanchez Petitto G., Sarwari N.M., Jain P., Swaby M., Bhattacharjee M. (2017). FDG PET/CT in Malignant Eccrine Spiradenoma. Clin. Nucl. Med..

[137] Shaw M., McKee P.H., Lowe D., Black M.M. (1982). Malignant eccrine poroma: A study of twenty-seven cases. Br. J. Dermatol..

[138] Coonley C.J., Schauer P., Kelsen D.P., Sordillo P., Huvos A.G. (1985). Chemotherapy of metastatic sweat gland carcinoma. A retrospective review. Am. J. Clin. Oncol..

[139] Bogner P.N., Fullen D.R., Lowe L., Paulino A., Biermann J.S., Sondak V.K., Su L.D. (2003). Lymphatic mapping and sentinel lymph node biopsy in the detection of early metastasis from sweat gland carcinoma. Cancer.

[140] Shiohara J., Koga H., Uhara H., Takata M., Saida T. (2007). Eccrine porocarcinoma: Clinical and pathological studies of 12 cases. J. Dermatol..

[141] Delgado R., Kraus D., Coit D.G., Busam K.J. (2003). Sentinel lymph node analysis in patients with sweat gland carcinoma. Cancer.

[142] Scrivener Y., Grosshans E., Cribier B. (2002). Variations of basal cell carcinomas according to gender, age, location and histopathological subtype. Br. J. Dermatol..

[143] Malone J.P., Fedok F.G., Belchis D.A., Maloney M.E. (2000). Basal cell carcinoma metastatic to the parotid: Report of a new case and review of the literature. Ear. Nose. Throat J..

[144] Jankovic I., Kovacevic P., Visnjic M., Jankovic D., Binic I., Jankovic A., Ilic I. (2011). Application of sentinel lymph node biopsy in cutaneous basosquamous carcinoma. Ann. Dermatol..

[145] Yoshida Y., Shiomi T., Tahira M., Yamamoto O. (2013). Metastatic basosquamous carcinoma detected by sentinel lymph node biopsy. J. Dermatol..

[146] Buffo T.H., Stelini R.F., Serrano J.Y.M., Pontes L.T., Magalhães R.F., de Moraes A.M. (2023). Mohs micrographic surgery in rare cutaneous tumors: A retrospective study at a Brazilian tertiary university hospital. An. Bras. Dermatol..

[147] Yesensky J. (2023). Sentinel Lymph Node Biopsy for Cutaneous Squamous Cell Carcinoma of the Head and Neck. https://clinicaltrials.gov/study/NCT05108090.

[148] Cives M., Mannavola F., Lospalluti L., Sergi M.C., Cazzato G., Filoni E., Cavallo F., Giudice G., Stucci L.S., Porta C. (2020). Non-Melanoma Skin Cancers: Biological and Clinical Features. Int. J. Mol. Sci..

